# “I can’t make all this work.” End of life care provision in natural disasters: a qualitative study

**DOI:** 10.1186/s12904-023-01137-0

**Published:** 2023-03-10

**Authors:** Marguerite Kelly, Imogen Mitchell, Iain Walker, Jane Mears, Brett Scholz

**Affiliations:** 1 School of Medicine and Psychology, Australian National University, 54 Mills Road, 2601 Acton, ACT Australia; 2 Melbourne School of Psychological Sciences, The University of Melbourne, Melbourne, VIC Australia; 3 School of Social Sciences, Western Sydney University, NSW, Australia

**Keywords:** Palliative care, End of life care, Life support care, Natural disasters, Epidemics, Pandemics, Patients, Caregivers, Health personnel, Qualitative study

## Abstract

**Background:**

Natural disasters are becoming more frequent and severe and profoundly impact the end-of-life care experience, including service provision. There is a paucity of research examining healthcare workers’ experiences in responding to care demands when disasters strike. This research aimed to fill this gap by exploring end-of-life care providers’ perceptions of the impact of natural disasters on end-of-life care.

**Methods:**

Between Feb 2021-June 2021 ten in-depth semi-structured interviews were conducted with healthcare professionals providing end-of-life care during recent natural disasters, COVID-19, and/or fires and floods. Interviews were audio-recorded, transcribed, and analysed using a hybrid inductive and deductive thematic approach.

**Results:**

The overarching theme from the healthcare workers’ accounts was of being unable to provide effective compassionate and quality care - “I can’t make all this work.” They spoke of the considerable burdens the system imposed on them, of being overextended and overwhelmed, having their roles overturned, and losing the human element of care for those at end-of-life.

**Conclusion:**

There is urgent need to pioneer effective solutions to minimise the distress of healthcare professionals in delivering end-of-life care in disaster contexts, and to improve the experience of those dying.

**Supplementary Information:**

The online version contains supplementary material available at 10.1186/s12904-023-01137-0.

## Background

Natural disasters are becoming more frequent and severe [1, 2], and exert profound impacts on healthcare professionals’ ability to deliver effective end-of-life and palliative care [3–7]. Natural disasters include: climatological (e.g. fire), meteorological (e.g. storm), geophysical (e.g. earthquake), hydrological (e.g. flood) and biological (e.g. epidemic/pandemic) disasters [8]. During natural disasters, end-of-life care service provision is strained and under-resourced, and care occurs across various care settings, including in specialist palliative care or hospice care, emergency and acute care [9, 10], aged care [11], or community care settings [12–15]. Healthcare workers may find themselves working in unfamiliar professional settings [16, 17], with their ability to deliver effective care impeded by shortages of material and human resources [18, 19]. They face additional pressures and strains, and encounter insurmountable challenges in managing uncertain end-of-life care trajectories [20–22], often with restricted support and involvement from patients’ family/social contacts [19, 20, 23, 24]. Within epidemics, healthcare professionals experience concurrent fears about catching and spreading the illness [18, 20], and may struggle to balance home and work commitments [15].

Effective communication between the healthcare professional and the dying patient, and also between the healthcare professional and the patient’s family members, is inevitably hampered [25, 26]. Healthcare professionals display resilience and commitment to their role [15, 17, 20], taking remarkable initiatives despite depleted resources and inadequate guidelines [6, 19], often assuming increased responsibility in patient care [25]. Studies report that healthcare professionals experience moral distress [27, 28], particularly when constraints leave them unable to deliver a quality of care consistent with their core values [29].

Despite the global reality of the increased frequency and severity of natural disasters, and recent instances of the provision of end-of-life care within these contexts, there is a gap in the research which examines healthcare professionals’ perspectives into the impacts of natural disasters on end-of-life care [3]. Logistical and practical challenges in conducting empirical research into end-of-life care impacts of unplanned or sporadic natural disasters have likely contributed to the scarcity of research [3]. The aim of this qualitative study is to improve understandings concerning healthcare professionals’ experiences in delivering end-of-life care within natural disasters at the actual time of crisis. In doing so we develop deeper understandings of health professionals’ unique experiences, to assist in the planning and preparation for future disasters.

## Methods

### Design, aim, and data collection

This study utilised a qualitative, exploratory design [30] to examine healthcare professionals’ perspectives on the impact/s of natural disasters on end-of-life care. Participants were recruited through social media, Facebook, and Linkedin, emails to end-of-life care services listed on the Palliative Care Australia website, and through snowball sampling. For inclusion in the study, participants needed to be aged 18 years and over and have had recent experience in the provision of end-of-life care during a natural disaster, COVID-19, bushfire, or flood. In-depth semi-structured interviews were undertaken by MK, an experienced qualitative researcher and counsellor, between February 2021 and June 2021. Sampling ceased when no new information was being generated in the interviews with regards to the themes identified here. Interviews ranged from 51 to 94 min, averaged 69 min, and were conducted in person (*n* = 2), online via Zoom (*n* = 5), or by telephone (*n* = 3) according to participants’ preference and capacity within the COVID-19 pandemic. Participants read and signed an informed consent form prior to the interview and received an AU$20 online gift card in recognition of their time.

The interview schedule was prepared during a series of discussions between the project team consisting of doctors, psychologists, and end-of-life care researchers. The questions were pilot tested, and the interview guide was refined to include emerging themes. Refer to Supplementary file 1 for the interview guide. No concerns arose during the pilot interview, so it was included in the final analysis. Open ended interview questions included ‘Tell me about your experience with a recent disaster’, and ‘Tell me how the disaster impacts on your role, your clients and the services you provide’. Follow-up questions were used as prompts during the interviews to explore the experiences of end-of-life service providers. The study was approved by the Australian National University Human Research Ethics Committee (2020/378). The reporting of this study is in accordance with the Standards for Reporting Qualitative Research (SRQR) checklist [31].

### Data analysis

Interviews were audio-recorded and transcribed by a professional transcriber. Transcripts were de-identified and pseudonyms used to refer to participants. MK undertook a thematic analysis following the phases outlined by Braun and Clarke [32]. A hybrid inductive and deductive approach was adopted, to allow theoretical underpinnings to remain integral to the analysis while remaining flexible in enabling new information to be explored directly from the data [33]. After repeatedly listening to the interview recordings and re-reading the transcripts, transcripts were systematically coded line-by-line to identify patterns in the data. Initial interviews were also coded by BS and IW to allow the team to discuss a broader scope of codes, and to develop a richer more nuanced reading of the data [34]. NVivo 12 software was used to assist with identifying and organising codes across the entire data set. Grouping relevant codes together and collapsing overlapping ideas, in an iterative manner, assisted in generating final themes.

## Results

### Characteristics of the sample

Ten end-of-life healthcare providers participated in the study (8 Female, 2 Male). Participants were aged 30–39 (*n* = 2), 40–49 (*n* = 5) or 50–59 (*n* = 3) and lived in urban (*n* = 5) or regional (*n* = 5) areas. Participants self-identified as Australian (*n* = 3), Asian (*n* = 1), Australian and Indigenous Australian/Aboriginal (*n* = 1), European (*n* = 2), Middle Eastern (*n* = 1), New Zealander (*n* = 1), and Irish (*n* = 1). At the time of their interview, they were working in Palliative Care (*n* = 2), Hospice (*n* = 2), Hospital (*n* = 3), Aged Care (*n* = 3), and Community (*n* = 3) settings. Three participants were working across multiple settings. Participants included doctors (*n* = 3), nurses (*n* = 2), psychologists/counsellors (*n* = 2), and those providing psychosocial support in end-of-life care services (*n* = 3). All participants were involved in the provision of end-of-life care during the COVID 19 pandemic: Two only in the COVID-19 pandemic, five in bushfires, two in floods, and one in both bushfires and floods.

### Findings

The overarching theme across the data was that healthcare workers interviewed felt ill-equipped to provide adequate end-of-life care within disaster contexts; *“I can’t make all this work.”* The four subthemes—as detailed in the subsequent headings—were that healthcare workers were overextended and overwhelmed, their roles were overturned, the human element of care was lost, and systems imposed unsustainable burdens.

#### Overextended and overwhelmed: “It was like a gorilla on our backs the whole time.”

End-of-life care providers described how the extra demands imposed by disasters left them feeling overextended and overwhelmed. The demands were far greater than the already stretched material and human resources could meet. They were required to extend their working roles and work in unfamiliar ways to accommodate the disaster.

In addition, informal support generally readily available, was disrupted. The support of familial carers and volunteers was impeded either because they were not allowed to visit healthcare facilities, or could not physically get around, due to road closures during floods, fires, or borders being sealed. The combination of these factors meant healthcare workers felt they were carrying the brunt of the system’s inability to deliver usual care:

*“I think we even suffered in our own way…thisguilt, of not being able to support them (patients) in the way that we know how.”*

Healthcare workers were overwhelmed while trying to balance their own personal disaster-related fears and simultaneously providing patient care. Many were also protecting their own families and homes from floods and bushfires. The fear of contracting COVID-19 was a constant and persistent source of stress throughout the pandemic. They were afraid that they would pass the virus on to patients or their family or friends. Healthcare workers explained that *“everyone was on edge”* and that *“it was like a gorilla on our backs the whole time”* to describe what it was like working under disaster conditions.

#### Roles overturned: “It was a complete 360 as to what I normally do.”

Healthcare providers found themselves doing work they were neither trained for nor had prior experience undertaking, for instance, organising the logistical responses to the disaster, and implementing outdated or non-existent disaster plans. For some, their scope of practice was expanded to include working with their local State Emergency Service to assist patients with access to food or medication, or for the transportation of the bodies of people who had died.

They were creative in the ways they responded when unable to meet people face to face, for example, utilising Telehealth, or connecting patients to other services when impacted by road closures. Taking on these additional responsibilities, also increased their administration loads, for instance in assisting families with exemption applications or putting in place and regulating COVD-19 visiting restrictions.

Healthcare workers described how role changes created difficult communication dynamics and strained relationships with the dying patient, or their family.

*“I ended up feeling like the bouncer. And that made it really difficult because people didn’t want to talk to you because you’re stopping their loved ones coming in the door. People weren’t engaged with me as much as they used to…‘Cause I saw less of them. And the times that I did see them were awkward and uncomfortable because it was about imposing restrictions.”*

Healthcare workers expressed discomfort over their changed roles, particularly when their roles shifted from working closely alongside dying patients and their families, to mandating restrictions. They felt ill-equipped to undertake the new role requirements and felt uneasy about the potential detrimental impacts on quality of care.

#### Losing the ‘human’ element when disaster strikes: “Everyone has the right to die with a full heart”

Healthcare workers described how the human element often got lost during the crisis, and this impacted patients’ end-of-life care experience:

*“There’s procedural plans. And the human connection can be lost, and the experience can become devastating…it needs to be about finding meaningful connections…within a crisis at the time*.*”*

Healthcare workers expressed concern that patients *“didn’t get the same care they would have if their family was there.”* Not having family and friends available left gaps in patients’ psychosocial and physical needs:

*“You do see people really settle when they have that family, that loved-one connection there. There was a lot more isolation, loneliness, even, issues with [sic] personal care. So maybe pads were left on longer.”*

*“People have stopped eating because they haven’t had their family there supporting them to eat.”*

Healthcare providers interviewed were distressed that some patients were dying alone and in isolation. They described the practice as *“totally insensitive and inhumane.”* Some stated that they would *“not like to die in these times”*, and that *“I don’t think it was always dignified.”* Healthcare providers understood how important advocacy was in achieving a suitable end-of-life care experience for patients. They described the importance of having significant people around dying patients:

*“There are particular points of our life, when we really need others…When we’re close to dying…that’s one of those periods.”*

*“Guidelines aren’t strong enough. It’s got to be policy so that people dying get the best end-of-life care they can, which…means having people around them, that are important.”*

Healthcare workers were also concerned that the experience of end-of-life care during the disaster would impact the grief process of those bereaved:

*“It will impact their memory of the death of their loved one…There will be a lot of people who will feel that they didn’t get to grieve properly because those…normal processes that we use socially to grieve…have been impacted…People… didn’t get to have… that really precious time with their loved one.”*

Healthcare providers described how there were fewer end-of-life conversations, and less opportunity for patients to complete what they needed to before they died:

*"There was much less of those conversations about their normal end-of-life care planning… in terms of, “Is your will in order?”, because either they were more distressed about their visitors not being able to come...Or it was too late to access the services being provided. Most businesses were closed down…it was difficult to get a lawyer, in a short amount of time."*

In addition, healthcare professionals reported that changes to routine care necessitated that more urgent care was often required later in the care trajectory:

*"Hospitalisation presentations very significantly decreased…Late diagnosis for heart attacks, and late diagnosis of cancer…were…much more common. So…not getting care as early as they would otherwise. I mean it’s hard to get palliative care involved early at the best of times. Certainly, it was made only harder...And so lots of really late presentations, really late involvement…high degrees of distress."*

Healthcare professionals reported that one of the key barriers to effective end-of-life care was inadequate access. Road closures due to fire or flood, and COVID-19 lockdowns restricted peoples’ access to care, family/friend support, and sometimes to food and medications. They described disasters as not only impacting patients’ quality of life and wellbeing, but also their place of death and dying experience.

A sense of not being able to fulfill holistic care needs within disaster settings was distressing for many healthcare workers interviewed. One healthcare worker described how others “*may actually have an even more important role than just…giving the paracetamol.”* There was an acknowledgment among those interviewed that even if medication needs were able to be addressed, there was a range of psychosocial elements equally important that were impeded by the disaster, including access to their loved ones, spiritual leaders and other supports.

#### Systems imposed unsustainable burdens: “None of this is a criticism of people”

Healthcare workers felt powerless in a system that imposed unsustainable burdens and rendered it impossible for them to perform the care processes that they understood as essential for patients at end-of-life. These healthcare workers were working with policies that *“change on the fly”*, often without warning or consultation. They had to navigate their way through often unclear or contradictory policies, wasting their time trying to figure out how to get their work done and falling short of their own care provision expectations:

*"I felt that I wasn’t doing a very good job because I wasn’t allowed to*.*"*

The imposition of new policies and rules and the shortage of resources meant they felt unable to do their usual work and instead struggled with administration and bureaucracy. Healthcare workers described how *“decisions were more weighted to the regulation safety, risk-averse side than caring for people at end-of-life with dignity.”* A sense of a lack of support on the frontline was a practical consequence of delayed or poorly executed disaster plans. Healthcare professionals described how more experienced senior staff often needed to focus on disaster response within the crisis, reducing further their access to effective workplace supports.

Disasters necessitated healthcare workers to function in a situation of care compromises. For example, there were delays in provision of basic care such as catheter care, feeding, and showering. It was difficult to maintain current care plus disaster requirements. They were often the ones delegated to reinforce the exclusion policies, rules, and procedures that they knew would inevitably lead to suffering for patients, family, and friends and cause moral distress for themselves﻿.

*"I don’t think that’s the fault of the staff. I believe it’s the fault of the system that’s set up in such a way that time constraints, resource constraints…impact the way that people die in a negative way."*

Despite this excerpt showing an awareness that systems were failing staff, the burdens added to staff were magnified, as they were aware that system failings would worsen the experiences of dying patients.

*Material and Human Resource Shortages.* Another key concern was the lack of access to human (staff) and medical resources such as syringe drivers, or adequate personal protective equipment (PPE). Some healthcare workers described how they purchased their own PPE: *“I bought my own masks.”* Staff shortages were of concern, especially access to staff adequately trained in end-of-life care. Some staff took leave because they personally had been impacted by the disaster, e.g., by smoke during bushfires, or to balance home care commitments during the pandemic, or due to their sense of fear and overwhelm. Providing care during a disaster with minimal resources and supports was a recipe for staff burnout. Some healthcare workers *“wanted to take leave and stay home until it’s all over.”*

*Healthcare Workers as Heroes.* The government, senior management, and media declarations of healthcare workers as ‘heroes’ compounded healthcare professionals’ moral distress, and their sense of responsibility and burden.

*“The heroicism [sic] of the…carer or the clinician…is quite problematic because…it… creates some kind of…value judgement about work that we’re essentially doing ‘cause it’s our job. But also it creates a…sense of…responsibility or an acceptance of particular risks… because one is in this heroic profession.”*

It is likely, though, that care provision was often the best possible based on the capacity and resources available:

*“Clearly, we have a responsibility to provide care based on the best availability of resources that we have, and the best capacity we have to do that. But we also…need to talk through…how that’s going to work, and what we can give, and what we can’t. What’s acceptable and what’s not.”*

This excerpt underscores the human element of the healthcare workers, who found themselves needing to confront and reconcile the limitations of care provision within the disaster context.

## Discussion

The findings of the current study demonstrate ways in which healthcare workers felt ill-equipped to provide adequate end-of-life care within natural disaster contexts. Healthcare workers’ ability to deliver effective care during natural disasters is challenged by their experiences of being overwhelmed, their roles being overturned, the disintegration of holistic elements of care, and by burdens imposed by the system.

Previous research has highlighted the additional barriers healthcare professionals encounter in delivering end-of life care during natural disasters, including because of resource shortages [18, 19]. The current study extends this knowledge, emphasising the psychosocial and system impacts. The present findings reflect a more nuanced representation of the synergistic nature of end-of-life care in disasters. That is, the impacts of disasters on end-of-life care service provision are compounded by the cumulative interplay of successive barriers. Disasters disrupt the synergistic nature of end-of-life care contributing to healthcare professionals’ sense that *“I can’t make all this work.”*

### What disrupts the synergistic nature of end-of-life care within natural disasters?

Although there is not one ultimate end-of-life care trajectory, a ‘good death’ is thought to include: receiving holistic end-of-life care; being treated with dignity and respect; not experiencing a sudden or unexpected death; prior preparation including advanced care planning; an awareness of impending death and opportunity for authentic communication [35]. The ‘good death’ theory posits that ‘on knowing the dying person’s preferences, all involved are to work towards achieving these; the place of death is important; the person’s family are involved and the needs of the bereaved are considered’ [35].

The healthcare professionals who participated in the current study reported a mismatch between the care they could ideally provide to facilitate a ‘good death’, and the care that was achievable within the disaster context. They described being unable to meet the holistic care needs of the dying patient and losing the ‘human’ elements of care. Healthcare workers experienced moral distress over their inability to connect patients with their friends or family, or other sources of support. Even if adequate medical resources were available health professionals believed that medication alone was not sufficient. Patients dying alone or in isolation, stripped of dignity and self-respect, was particularly distressing for healthcare workers. Fewer end-of-life conversations lead to gaps in psychosocial, spiritual, and existential care needs, and compounded grief for families and friends. Future research should continue to explore how humanistic, compassionate, and holistic elements of end-of-life care can be sustained despite barriers imposed by disasters.

Several recent studies mirror these findings. Costantini et al. [15] found hospice staff raised concerns over an inability to provide holistic care within the constraints of the disaster. Franchini et al. [25] described how health professionals experienced an overwhelming sense of responsibility for patient care. This was particularly the case when there was inadequate access to essential resources and when friends and family were absent because of visitation restrictions, lockdowns, or road closures. Franchini et al. [25] and Tavares et al. [26] reported many instances of hindered communication between health professionals and patients or their families and friends. Our study extends these findings, by demonstrating how shifting roles (from working in partnership with the consumers/service users to imposing regulations) exacerbate these communication problems.

Watson’s human caring theory within the context of palliative care emphasises the human elements of care, and the importance of sharing care between close significant others and healthcare providers [36]. Our study found that disasters disrupted both the ability to deliver the human elements of care, and the ability to deliver care in partnership with relatives and friends. Many healthcare workers responded by subsuming increased responsibility, while others considered remaining at home until the disaster was over, particularly if they were experiencing their own disaster-related fears. The inability to share care with patients’ close significant others is both unsustainable for healthcare professionals, and directly opposes the definition of a ‘good death’ [35].

Consistent with recent research [27–29], healthcare professionals within the present study experienced moral distress concerning adequate care provision. Their moral distress arose from: being overextended and overwhelmed with limited resources and workplace supports, being unable to deliver holistic care or care consistent with their values, and in response to burdens imposed on them by health systems. The present study demonstrated how disasters tip an already strained system into crisis, negatively impacting the experiences of healthcare professionals and dying patients.

### Strengths and limitations

This qualitative study extends the knowledge concerning the impacts of natural disasters on end-of-life care, from the perspective of healthcare professionals. The analysis of the interviews explores the in-depth experiences and challenges of healthcare professionals directly involved in the provision of end-of-life care in recent natural disasters. Future research could explore the experiences of other first line disaster responders, for example, police force workers, armed force workers, or fire fighters, with a view to greater collaboration between healthcare and non-healthcare organisations within disasters. It is a strength of the study that participants who were operating under extreme stress in time-poor, chaotic work conditions, desired to provide valuable time to participate in the study.

The study is not without limitations. The study took place during the COVID-19 pandemic which may have shaped participants’ overall experience. However, participants had opinions about and experienced a range of disaster types, thus the analysis is not limited to the pandemic context. It is a strength of the present study that not only were health professionals treating patients impacted by disasters, but they were also experiencing the stress and trauma of being part of and affected by these disasters - a complex, platform in which to deliver care. The study was undertaken at a critical time in the COVID-19 pandemic when community fear was heightened, vaccination had only just commenced, and Rapid Antigen Tests were not yet available. This study provides an important snapshot and can act as a comparison for future research.

### Contributions and implications of this study

This study begins to address the research gap examining the impacts of natural disasters on provision of end-of-life care by healthcare professionals. The study found that within disasters healthcare professionals are significantly challenged in delivering care consistent with the underlying assumption of a ‘good death’, or in accordance with Watson’s human caring theory, or indeed in accordance with the very definition of palliative care. As well as its usefulness in describing current findings during a time of crisis, the present research serves as a comparison for future work, to gauge progress of end-of-life care in disasters. The findings can also assist in the planning and preparation for future disasters.

The present research has clear clinical and policy implications. Health services offering end-of-life care need to ensure that workers have access to adequate training and support concerning disaster response. Providing psychosocial support for healthcare professionals is crucial to combat potential moral distress and burnout. Similarly, finding ways to bolster psychosocial care for patients and address their holistic care needs within disasters is of critical importance. End-of-life care conversations are an area that require ongoing focus in both research and clinical practice, as essential features within disaster contexts. Ensuring effective communication between the healthcare professional and the patient and/or family about when death is anticipated, would be an important first step to beginning to address holistic care needs.

Outsourcing additional bureaucratic and administrative tasks during disasters to others not serving on the front line and bolstering the workforce with volunteers trained in psychosocial support, disaster response and use of personal protective equipment is an avenue that could be explored in preparation for future disasters to help take pressure off healthcare workers. Providing necessary human and material resources and enough flexibility within health systems to allow end-of-life care professionals to undertake their important work is critical.

## Conclusion

This study examined the impacts of natural disasters on end-of-life care from the perspective of healthcare professionals. The current findings serve as comparison for future research and will assist in the planning and preparation for future disasters. The recent societal framing of healthcare workers as heroes who must soldier on despite all the pressures and with limited support is problematic and fails to recognise the human elements implicit in healthcare workers themselves. This study highlights the experience of moral distress among end-of-life care providers, that may lead to burnout, at a time when the COVID-19 pandemic continues, and care demand remains high. There is urgent need to pioneer more effective solutions to minimise healthcare professionals’ distress in delivering end-of-life care in disaster contexts, and to improve the experience of dying patients and their friends and families.

## Electronic supplementary material

Below is the link to the electronic supplementary material.

Supplementary Material 1

## Data Availability

Our ethics approval does not extend to making data freely available, but we are happy for interested parties to contact us should any clarification be sought. Data can be available from the corresponding author on reasonable request.
