# Supplementary material for: “I can’t make all this work.” End of life care provision in natural disasters: a qualitative study
Source: BMC Palliat Care. 2023 Mar 10;22:21. doi: 10.1186/s12904-023-01137-0 (PMC9999053; doi:10.1186/s12904-023-01137-0)
Supplement: Supplementary file 1 — Supplementary Material 1 [file 12904_2023_1137_MOESM1_ESM.pdf]

Supplementary File 1. Interview guide

- Tell me a bit about your role in working with people with life-limiting, terminal, or life threatening illness?
- Tell me about your experience with a recent disaster (e.g., bushfire, COVID-19, or flood) and how it may have impacted care for/or the care experience of someone with life-limiting, terminal, or life-threatening illness?
- Did the disaster impact your role, your clients or the service provided? (If yes). Tell me how the disaster impacts on your role, your clients, and the services you provide?
- Do/did you draw on any policy/guidelines in the provision of care in these contexts? (If yes, how?)
- Did the disaster necessitate any changes to how things may have been experienced generally?
- Do you feel professionally prepared to operate in disaster environments?
- Did you experience any constraints/limitations on your ability to act in such circumstances?
- What would you change (if anything) to improve the experience for those providing care, or receiving care during disaster contexts?
